# Variation in Response to Water Availability Across *Phlox* Species

**DOI:** 10.1002/pei3.70098

**Published:** 2025-11-08

**Authors:** Christina Steinecke, Julius A. Tabin, James Caven, Charles O. Hale, Antonio Serrato‐Capuchina, Robin Hopkins

**Affiliations:** ^1^ Department of Organismic and Evolutionary Biology Harvard University Cambridge Massachusetts USA; ^2^ Arnold Arboretum of Harvard University Roslindale Massachusetts USA; ^3^ Section of Plant Breeding and Genetics Cornell University Ithaca New York USA; ^4^ Department of Biology Boston College Boston Massachusetts USA

**Keywords:** ecological differentiation, hybridization, life history, niche modeling, phenotypic plasticity, *Phlox*

## Abstract

Plants adapt to environmental variation both by evolving divergent trait means and by plastically adjusting trait expression in response to local conditions. While these dual strategies are essential for persistence in diverse environments, there are still outstanding questions about how they interact and vary across closely related species. For plants, water availability is a particularly important selective force that shapes species distributions, selects for growth habit and life history strategy, and can dictate individuals' plastic expressions of trait values and reproductive success. Here, we use ecological niche modeling, field soil characterization, and a controlled dry‐down experiment to understand how geographic distribution and evolutionary background among three closely related *Phlox* wild flower species and their F1 hybrids explain their responses to water availability. We infer that the species occupy distinct niches that diverge along a primary axis of water availability and soil moisture. Each species has a distinct growth habit that does not match broad predictions of divergence in response to water availability. Nevertheless, we find that all the species show a significant morphological response to controlled soil dry down with reduced biomass, smaller leaves, and fewer flowers, as would be predicted in a response to drought. We find that *Phlox drummondii* , which occupies intermediate habitats, exhibits the strongest plastic response to water limitation, despite it not having the broadest environmental niche. Additionally, most hybrids involving *P. drummondii* display intermediate phenotypes in both wet and dry treatments, while hybrids between *P. cuspidata* and *P. roemeriana* show phenotypes consistent with hybrid vigor. These results challenge the hypothesis that species from broader environments evolve greater plasticity. Instead, the most plastic species did not have the broadest niche, suggesting plasticity and niche breadth may evolve independently.

## 1 Introduction

As sessile organisms, plants must adapt to and grow in the environment in which they germinate. Two distinct processes allow them to do so. Trait divergence reflects evolutionary history and heritable differences in mean trait values that accumulate between lineages adapted to different environments (Bohnert et al. 1995; Kawecki and Ebert 2004; van Kleunen and Fischer 2005; Anderson et al. 2011; Stotz et al. 2021; Vinton et al. 2022). In contrast, phenotypic plasticity is the capacity of an individual (or genotype) to produce different phenotypes depending on the environment it experiences and to respond to environmental heterogeneity (Bradshaw 1965; Via et al. 1995; Roff 1999; Agrawal 2001). Both divergence and plasticity influence plant performance in nature, but they can operate on different time scales, with trait divergence occurring over evolutionary timescales and phenotypic plasticity occurring within a single generation (Anderson et al. 2011; Stotz et al. 2021). Although much work has focused on trait divergence and plasticity separately, relatively little is known about how the two interact to shape environmental response, particularly across closely related species (but see Ghalambor et al. 2007; Pfennig et al. 2010; Husemann et al. 2017).

Phenotypic plasticity broadly refers to an organisms ability to adjust the expression of a trait, whether physiological, developmental, morphological, or behavioral, in response to environmental conditions (West‐Eberhard 1989; DeWitt and Scheiner 2004). Contrastingly, evolutionary divergence refers to genetic differentiation between lineages that results in heritable trait differences over evolutionary time. Plasticity often acts as a “first responder,” allowing individuals to adjust within their lifetime and survive in new or fluctuating environments without waiting for evolutionary change (Chevin et al. 2010; Chevin and Lande 2010; Snell‐Rood et al. 2018). When selective conditions are consistent, initially plastic traits may become genetically assimilated and canalized leading to evolutionary differentiation (Agrawal 1999; Chun et al. 2007; Wund et al. 2008).

The degree, or magnitude of plasticity can vary widely even among closely related individuals, populations, and species (Wund et al. 2008; Nielsen and Papaj 2022; Vinton et al. 2022; Walter et al. 2022; de la Mata and Zas 2023). In much the same way that heritable trait differences evolve and respond to selection from the environment, the degree of plasticity can also evolve and therefore vary across organisms. It has been hypothesized that species which inhabit broad abiotic or biotic environmental conditions that show high levels of stochasticity will have greater plasticity than species that are restricted to specific environmental conditions (Pfennig et al. 2010; Leung et al. 2020; Stotz et al. 2021). In these circumstances, a species can express a wide variety of trait values in response to the breadth of environmental conditions it may encounter.

Furthermore, it has been hypothesized that this ability to plastically respond to many environments allows a species the time and opportunity to evolve adaptations to these environments (West‐Eberhard 1989; Ghalambor et al. 2007). This pattern of genetic adaptation following initial plastic responses to environments will lead to a pattern in which the dominant axis of genetic divergence in response to environmental variation aligns with the direction of plastic response to the environmental variation (Lind et al. 2015; Diamond and Martin 2016; Walter et al. 2022). In other words, the plastic response of a species to an environmental stimulus is hypothesized to be in the same direction as trait divergence across closely related species that are adapted across the same environmental gradient (de Jong 2005; Diamond and Martin 2016; Radersma et al. 2020). For instance, in two closely related *Senecio* species distributed along an elevational gradient, *S. chrysanthemifolius* (low elevation) and *S. aethnensis* (high elevation), it was found that trait divergence corresponded closely with plastic responses expressed when each species was grown across the elevational range (Walter et al. 2022). A number of processes shape the extent of plastic responses in nature. For example, ecological context can influence the evolution of plasticity (Kulkarni et al. 2011; Kellermann et al. 2018), evolutionary history may constrain or facilitate plasticity (Pigliucci et al. 1999; Kellermann et al. 2018), and environmental stochasticity can alter the predictability of selection, thereby shaping the adaptive value of plasticity (Leung et al. 2020).

Much can be inferred about the evolution of plasticity and adaptive divergence by comparing closely related species and even more can be learned by also examining their hybrids. Hybridization between lineages that differ in adaptive trait means or in plastic responses to environmental conditions could result in hybrid individuals with either intermediate or extreme (transgressive) trait means or plasticity (Rieseberg et al. 1999; Stelkens and Seehausen 2009; Dittrich‐Reed and Fitzpatrick 2012; Husemann et al. 2017). These two alternative outcomes could suggest different evolutionary paths. Intermediate trait means in hybrids suggest species differences are due to additive genetic variation, consistent with evolution from shared ancestral variation that does not interact with strong negative or positive epistasis (Stelkens and Seehausen 2009; Hill et al. 2008; Mackay 2014; Tan et al. 2018). Similar inferences could be made about additive genetic variation underlying intermediate plasticity in hybrids (Hill et al. 2008; Mackay 2014). In hybrids, transgressive traits are hypothesized to emerge when parental species evolve novel genetic variation across multiple loci or pathways in isolation that either complement each other's phenotypic effects or interact with epistasis in hybrids (Rieseberg et al. 1999; Reiseberg et al. 2003; Stelkens and Seehausen 2009; Kagawa and Takimoto 2018). In this way, investigations into the environmental responses of hybrid lineages can be a tractable way to gain insight into the evolution of environmental responses and the reproductive barriers that limit hybrid establishment.

Water availability is one of the most important abiotic environmental determinants of plant success. Variation in water availability across time and space significantly contributes to regulating species presence, absence, and range limits (Eckstein 2005; van Kleunen and Fischer 2005, and Liu et al. 2017). Furthermore, there are countless studies documenting the plastic response to drought across all scales of biological organization from gene expression to developmental timing, to morphology (reviewed in Des Marais et al. 2013). There is extensive literature on the response of model and agricultural species to drought (El Hafid et al. 1998; Bartlett et al. 2016; Hoover et al. 2017), but we know much less about how drought response evolves in both non‐model species and across closely related species. Plants have likely evolved numerous unique responses to mitigate drought stress, including genetic (Monroe et al. 2018), physiological (Foulkes et al. 2007; Gupta et al. 2020), and phenotypic (Branch et al. 2024) adaptations. Typically, drought response manifests as alterations in overall growth, membrane integrity, photosynthetic activity, and water‐use efficiency (Hoover et al. 2017; Gupta et al. 2020). Experimental comparative studies of species across moisture gradients thus could have the potential to further tease apart how species adapt to drought stress and the underlying genetics of these drought response traits.

**FIGURE 1:**
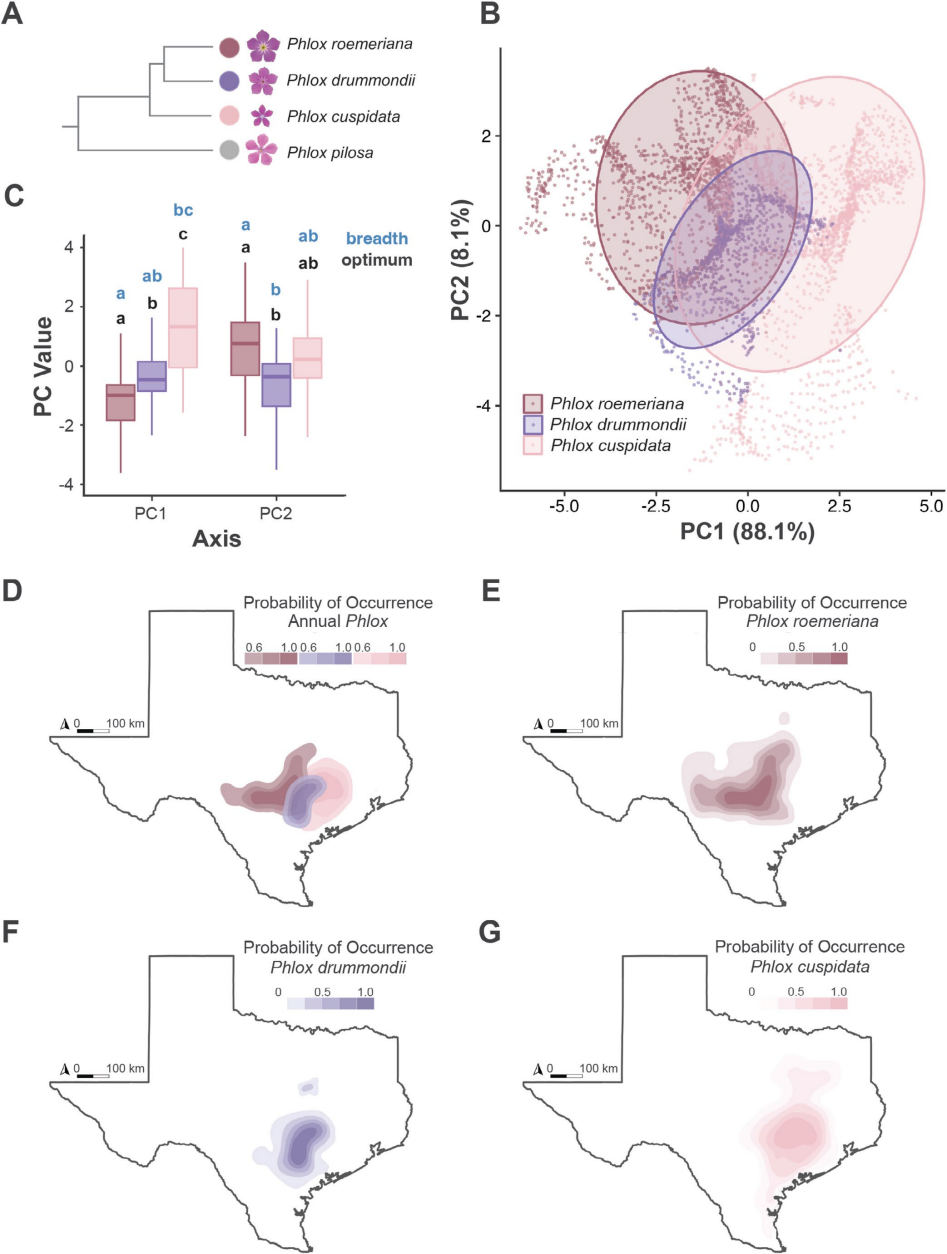
Characterization of niche for *Phlox cuspidata* (light pink), *Phlox drummondii* (purple), and *Phlox roemeriana* (dark pink). (A) Schematic of the phylogenetic relationship between Texas annual *Phlox*, adapted from Garner et al. (2024). (B) First two principal components of a PCA with projected distributions and WorldClim environmental variables, where each point represents a single pixel in our distribution model. (C) Optimum and breadth of principle components from PCA of projected species distributions. Comparisons are made across taxa, where niche optimum and breadth correspond to the median and length of the 95% inter‐percentile interval along the two PCA axes respectively. Unique lettering refers to a significant difference between species in niche optimum and/or breadth, *p* < 0.05. (D) Overlapping projected distributions of Texas annual *Phlox*, where probability of occurrence > 0.5. (E) Projected distribution of *Phlox roemeriana* . (F) Projected distribution of *Phlox drummondii* . (G) Projected distribution of *Phlox cuspidata* .

*Phlox drummondii* , *P. cuspidata* , and *P. roemeriana* comprise a small clade of three annual herbaceous flowering plant species native to Texas, USA. These three species have recently diverged (~2 mya), with *P. cuspidata* being an outgroup to sibling taxa *P. drummondii* and *P. roemeriana* (Roda et al. 2017; Garner et al. 2024; Figure 1A). Among these, *P. drummondii* occupies a large central range, overlapping with *P. cuspidata* in the east and *P. roemeriana* in the west, while *P. cuspidata* and *P. roemeriana* do not overlap in their distributions (Roda et al. 2017). These species are butterfly‐ and moth‐pollinated (Burgin et al. 2023), and can be found in prairie remnants, pastures, and roadside ditches. Little is known about the specific abiotic factors distinguishing the habitats of these species; however, *P. roemeriana* has been described as specialized to more xeric and calcareous soils relative to both *P. cuspidata* and *P. drummondii* , which are both found in similarly loamy habitats (Erbe and Turner 1962; Levin 1978a). Furthermore, unlike *P. roemeriana* and *P. drummondii* , *P. cuspidata* is self‐compatible and produces abundant selfed seed in the field (Levin 1978a, 1978b; Roda and Hopkins 2018; Shahid et al. 2024). Differences between these species are also reflected in their genomic data, with *P. drummondii* having the highest heterozygosity, effective population size, and overall genetic variation (Levin 1978b; Wu et al. 2025 unpublished data). Despite genomic evidence of gene flow across species (Roda et al. 2017; Garner et al. 2024; Wu et al. 2025 unpublished data) and interspecific compatibility in greenhouse settings with controlled crosses (Suni and Hopkins 2018), F1s among species pairs are only documented in low frequency in the field (Hopkins and Rausher 2012; Hopkins et al. 2014; Wu et al. 2025 unpublished data). Given the overlapping ranges with areas of sympatry, close relatedness, varied evolutionary histories, and ongoing gene flow between these species, Texas annual *Phlox* provide an excellent system for analyzing the causes and extent of trait divergence and plasticity to environmental stimuli, as well as their effects. In this study, we compared water availability for *P. cuspidata* , *P. drummondii* , and *P. roemeriana* using ecological niche modeling and soil analysis, and examined how these factors influence the potential for phenotypic plasticity under controlled drought conditions. Specifically, we ask: (1) How do life history differences, relatedness, and geographic distribution among *Phlox* species influence the direction and magnitude of phenotypic plasticity in response to drought? (2) How are these responses expressed in F1 hybrids, and what do they reveal about the genetic basis of phenotypic plasticity? Because *P. drummondii* harbors the most amount of genetic variation and has been described as occupying the largest range (Erbe and Turner 1962; Levin 1978a; Roda et al. 2017), we expected it to exhibit the highest phenotypic plasticity. At the same time, we predicted that both *P. cuspidata* and *P. roemeriana* would tolerate drought stress, given their mating system and specialization to xeric habitats, respectively. For the hybrids, we expected intermediate responses if phenotypic plasticity is primarily governed by additive genetic variation. Alternatively, if parental lineages have evolved divergent mechanisms of drought response, we expected hybrids to show either reduced plasticity (due to genetic incompatibilities) or transgressive responses that exceeded parental trait ranges.

## 2 Methods

### 2.1 Niche Modeling

We characterized the environmental niches of each of the three annual Texas *Phlox* species using species distribution modeling. All analyses were conducted in R version 4.3.3 (R Core Team 2024). We acquired population location data from across the ranges of the focal species based on records from our recent field collections and supplemented with distribution records from the Global Biodiversity Information Facility (GBIF; downloaded 10 December 2023, www.Gbif.org). We filtered GBIF occurrences to include only locations with verified images. This provided 324, 180, and 296 total locations for *P. drummondii* , *P. cuspidata* , and *P. roemeriana* , respectively (*N* = 800). We thinned these occurrence points to one location for every five‐kilometer grid cell to reduce sampling bias using the *thin* function from the “sp” package version 2.1–1 (Bivand et al. 2008). We further reduced our dataset to 180 populations per species (*N* = 540) for consistent modeling across species. To characterize potentially uninhabitable sites, we created 1000 pseudoabsences of each species by using the *randomPoints* function from the “dismo” package version 1.3–14 (Hijmanset al. 2023).

We extracted environmental variables from the WorldClim database (downloaded 10 December 2023; www.WorldClim.com/version2) using the *getData* function from the “raster” package version 3.6–26 (Hijmans and van Etten 2012). Layers were trimmed and aligned to include only the state of Texas (latitude 25.5°–35°; longitude −104° to −94°), representing an area of intermediate size that the focal taxa likely had the opportunity to disperse into (Sobel 2014). We selected seven layers based on their weak correlations with other layers (|*r*| < 0.7) and high importance to distribution models using the function *select07* from the “mecofun” package version 0.5.1 (Dormann et al. 2013; Table S1). Variables were consistent among species and included three temperature and four precipitation layers at 2.5‐min resolution.

We used the *maxent* function from the “Maxent” package version 3.4.0 (Phillips et al. 2006; Elith et al. 2011) to generate individual distribution models for each species. Maxent compares the distributions of environmental variables at sites occupied by a focal species to the distributions of pseudoabsences (Phillips et al. 2006; Elith et al. 2011). We used the *kfold* function from the “dismo” package to cross‐validate the distribution model of each species, as well as the *evaluate* function from the “dismo” package to calculate the area under the receiver operator curve (AUC). The AUC is a threshold‐independent indicator of species distribution model performance that ranges from 0.5 to 1, where a value of 0.5 indicates that a model is equivalent to a random draw and a value of 1 indicates that a model perfectly predicts the suitable habitat of a species (Phillips et al. 2006; Sobel 2014).

To assess differences in habitat among species we used three separate approaches. First, we filtered the distributions of each species to include only pixels/locations of populations that had a probability of occurrence > 0.5. We extracted from the environmental layers these predicted distributions of each species and used a Principal Component Analysis (PCA) to visualize the difference in habitat, niche breadth (the width of the environmental range tolerated by a species), and niche optima (the mean environmental variable value at the location with the highest predicted suitability) of each species. We then analyzed this pixel data using a one‐way Multivariate Analysis of Variance (MANOVA) to construct 95% confidence intervals, as described in Sobel and Streisfeld (2015). We performed post hoc analyses on each of the seven environmental variables using the *emmeans* function from the “emmeans” package version 1.10.0 (Lenth et al. 2024) to determine pairwise differences in species distributions. Finally, we used the *nicheOverlap* function from the “dismo” package on pairs of species to determine Schoener's D, quantifying the extent to which a pair of species may interact in the same space. Schoener's D ranges from 0 to 1, with a value of 0 indicating no overlap in predicted species distributions and a value of 1 indicating complete overlap in predicted species distributions (Schoener 1968).

### 2.2 Soil Characterization

We characterized soil properties in which the three *Phlox* species grow using samples collected over a single week in May 2023 from five populations representing the range of each species (a standard approach in comparative ecological studies; e.g., Yang et al. 2022). To ensure samples represented each population, we collected soil from 10 to 15 random sites within each population. At each site, we first cleared surface litter from the site and estimated soil moisture in parts per million with a moisture probe (Atree Soil Tester, Model no. B0DRFYCTYG). We then used a spade to dig a 6‐in.‐deep V‐shaped hole and collected a 1‐in. × 1‐in. core from the spade. Cores from all sites within a population were combined to create a population composite sample in a 1‐pint plastic bag (*N* = 5 composite samples per species or 15 total composite samples), which was left open to air dry for at least 24 h. We sent samples to the Soil, Water, and Forage Testing Laboratory at Texas A&M AgriLife (College Station, TX) to estimate a suite of chemical and physical traits including micro and macro nutrients, proportion of organic matter, and particle composition. For each variable measured, we ran an Analysis of Variance (ANOVA) test comparing the three species, using base R's *oneway.test* function. We also used the *cor* function to determine the Pearson correlation coefficient between each variable. Finally, we performed a PCA to examine patterns of overall differentiation among species in soil properties.

### 2.3 Drydown Experiment

We performed a controlled dry‐down experiment to characterize how the growth habits of the three Texas *Phlox* species and their hybrids change in well‐watered and dry conditions, and the differences in plasticity—response to drying—across the clade.

#### 2.3.1 Experimental Seeds

Seeds of each species were collected across their native ranges in Texas in May 2019 to ensure representative sampling of each species. We used seeds from 10 *P. drummondii* , 8 *P. cuspidata* , and 4 *P. roemeriana* populations (Table S2). The wild‐collected seeds were grown under controlled conditions in the greenhouses at the Arnold Arboretum of Harvard University (Boston, MA, USA) and used to generate experimental seeds from within‐population half‐sib families using controlled crosses. Crosses were performed within species, as well as between species to generate hybrid seeds. To induce germination, all seeds were soaked in 500 ppm Gibberellic Acid for 48 h and cold stratified for 7 days. Germination occurred in seedling trays filled with LM‐GPS Germination Mix (Lambert, Quebec, Canada) seedling potting media in controlled growth chamber conditions with 16 h of supplemental light at 23°C and a nighttime temperature of 18°C.

#### 2.3.2 Wet and Dry Treatments

We transplanted 361 germinated seedlings (1–16 individuals from each seed family from each species) 2 weeks after germination into 4‐in. square pots containing 200–300 g of Promix High Porosity Mycorrhizae soil. Due to low germination success, our sample sizes were limited such that *P. drummondii* had 23 seed families with 133 individuals; *P. cuspidata* had 22 seed families with 92 individuals; *P. roemeriana* had 14 seed families with 71 individuals; *P. cuspidata–P. roemeriana* hybrid had 8 seed families with 24 individuals; *P. drummondii–P. cuspidata* hybrid had 6 seed families with 35 individuals, and *P. drummondii–P. roemeriana* hybrid had 1 seed family with 6 individuals. Plants were fully randomized and distributed across 38 trays in the greenhouse. Individuals were allowed to acclimate for 1 week with a standard watering regime before being assigned to a treatment. Within each seed family, siblings were split into the dry and wet treatment groups to ensure genetic representation across the two treatments (*N* = 177 in dry, 183 in wet).

Wet and dry soil treatments were enforced by controlling the percent soil saturation of each pot. Wet treatment pots were maintained at 70% soil moisture whereas dry treatment pots were maintained at 12% soil moisture (percentages modified from Suni et al. (2020) due to humidity conditions in the greenhouse). Previous trials with *Phlox* plants indicate that 70% soil saturation corresponds to well‐watered pots and healthy flourishing plants, whereas 12% soil moisture results in plants that can survive but appear water stressed. Both treatments consisted of weighing and watering individual pots three times each week to reach target soil saturation levels. Soil saturation for each pot was calculated as

$$\begin{array}{c} \text{target weight}=\left(\mathrm{wet}\;\text{weight}–\mathrm{dry}\;\text{weight}\right)\times \text{target saturation percentage}\\ \;+\mathrm{dry}\;\text{weight} \end{array}$$

where dry and wet weight of each pot was measured at the start of the experiment prior to transplanting plants into pots. Dry weight was defined as the weight in grams of a pot filled with dry potting media, and wet weight was defined as the weight of the pot with potting media that is saturated with water. Individuals and their pots were weighed and watered according to their assigned treatment for 5 weeks.

For each individual, we counted the days from treatment to the opening of the first flower and collected four flowers within 10 days of first flowering. We scanned (EPSON V600) each flower to measure mean flower tube length and mean flower area in ImageJ (Schneider et al. 2012; www.ImageJ.net). At 9 weeks after germination, individuals were harvested. Upon harvesting, height, the longest leaf length, leaf count, and flower count were measured. We also estimated above ground wet biomass in grams by harvesting all stem, leaf, floral and fruit tissue growing above the surface of the potting media and quantifying weight on a balance. We estimated dry biomass in grams by placing the tissue in a drying oven for 3 days before weighing it again.

#### 2.3.3 Analyses

We summarized the phenotypic variation across all the traits using a PCA on the pure species in Scikit‐learn. We used the PCA loadings to determine which traits explained the variation along the dominant axes.

We performed a dimensionality reduction on the nine phenotypic traits for the three focal species in Python version 3.11.6 (Van Rossum and Drake 2009), using the package umap‐learn version 0.5.5 (McInnes et al. 2018). To do this, a standard scaler was used on the data from the package scikit‐learn version 1.3.0 (Pedregosa et al. 2011).

To quantify the response for each trait of the species to water limitation we performed a mixed‐effect linear model with the function *lmer* from the package “lme4” version 1.1.34 (Bates et al. 2015). We tested for an effect of species (excluding hybrids due to small sample sizes), wet/dry treatment, and the interaction across the nine phenotypic traits. Treatment, species, and their interaction were treated as fixed effects, while block effects and maternal family ID were included in the models as random effects. Paternal effects were excluded as a random effect, as they were found to be redundant with maternal family ID and led to overfitting. The fixed effects of each model were analyzed using an ANOVA test from the package “car” version 3.1.2 (Fox and Weisberg 2019) with Bonferroni correction, followed by a post hoc Tukey HSD test.

For all species and hybrids, we calculated normalized mean values for each trait by converting raw values to *z*‐scores to facilitate comparison across traits and groups.

## 3 Results

### 3.1 Niche Modeling

Our species distribution models predict the suitable habitats of the three species, as indicated by high AUC values (> 0.95; Table S3). These models reveal that the three taxa occupy geographically overlapping but distinct habitats (Figure 1B–G) across an east–west gradient, with *P. cuspidata* predicted to occur in the wettest and warmest habitats, *P. drummondii* occupying a moderate habitat, and *P. roemeriana* growing in the driest and coolest habitats (Table 1; Table S4). Overall, the species have significant differences in both niche optima and niche breadth along PC1 and PC2 (Figure 1B,C; Table 1). In fact, each species pair inhabits significantly different conditions across each environmental variable used in our species distribution modeling (Figure S1; Tables S5 and S6). The values of pairwise Schoener's D reflected the same trend, with the distributions of *P. drummondii* and *P. cuspidata* overlapping the most (0.77) and *P. roemeriana* and *P. cuspidata* overlapping the least (0.56). When we identified the most likely habitat of each species and isolated the WorldClim environmental variables associated with each of the species' occupied space pixels, we found that environmental variables related to precipitation (precipitation of the driest quarter, precipitation of the coldest quarter, and precipitation of the wettest quarter) explained 88.1% of the variation in PC1. Thus, soil moisture distinguishes the species' distributions (Figure 1C; Table S7).

**TABLE 1:** Niche optima of each species, where values represent the means of WorldClim variables in locations with the highest predicted suitability.

Variable	Environmental description	Niche optima value		
		*P. roemeriana*	*P. drummondii*	*P. cuspidata*
Bio7	Temperature annual range (°C)	30.0	32.5	32.0
Bio8	Mean temperature of warmest quarter (°C)	23.1	23.6	23.9
Bio11	Mean temperature of coldest quarter (°C)	10.5	10.6	11.1
Bio13	Precipitation of wettest month (mm)	106.0	117.0	127.0
Bio15	Precipitation seasonality	30.0	34.0	30.0
Bio17	Precipitation of driest quarter (mm)	149.0	160.0	198.0
Bio19	Precipitation of coldest quarter (mm)	149.0	165.0	204.0

### 3.2 Soil Testing

**FIGURE 2:**
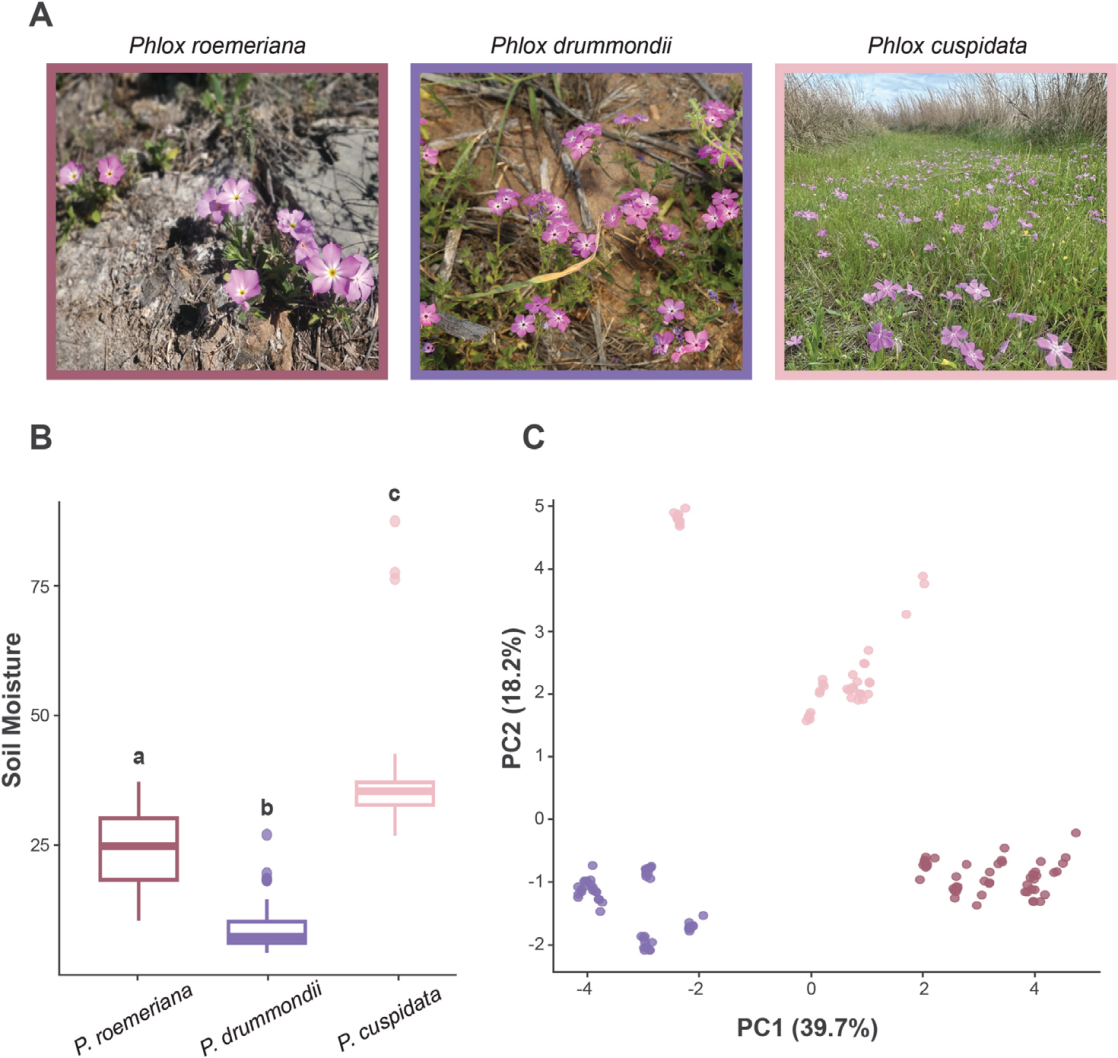
Differences in soil characteristics of *Phlox cuspidata* (light pink), *Phlox drummondii* (purple), and *Phlox roemeriana* (dark pink). (A) Photographs depicting typical habitats of *P. roemeriana* (left), *P. drummondii* (center), and *P. cuspidata* (right). Photos by Patrick Alexander (https://www.inaturalist.org/observations/265914863), Victor L. Manuel (https://www.inaturalist.org/observations/281151460), and Iuisesc (https://www.inaturalist.org/observations/270333215) via iNaturalist. Licensed under CC BY‐NC 4.0. (B) Differences in moisture (ppm) of soil samples from natural habitats. Comparisons were done using an ANOVA test with Bonferroni correction (unique lettering indicates *p* < 0.05). (C) Plot of the first two principal components for *P. cuspidata* , *P. drummondii* , and *P. roemeriana* based on soil samples, colored by species. See Table S10 for PCA loadings.

We found substantial variation across the soil properties across species (Figure 2A–C). Each sampled metric showed significant differences across species (*p* < Bonferroni‐corrected *p*‐value < 0.05; Table S8). Soil moisture is also significantly different between the three species (Figure 2B; Table S8) with *P. cuspidata* 's environment containing higher moisture than that of *P. roemeriana* (*p <* 0.001) and *P. roemeriana* 's environment containing higher moisture than that of *P. drummondii* (*p* < 0.001). Many of the soil parameters were highly correlated with one another (Table S9), and strongly species specific, as evidenced by tight species clustering in the PCA (Figure 2C; Table S10).

### 3.3 Drydown Experiment

To assess response to water availability and soil moisture in Texas *Phlox*, we performed a controlled drydown experiment where each species was grown in both dry and well‐watered conditions. We evaluated constitutive differences between species as well as responses of each species to variation in conditions using both multivariate and univariate analyses of phenotypic measurements.

#### 3.3.1 Multivariate Response to Water Availability Across Species

**FIGURE 3:**
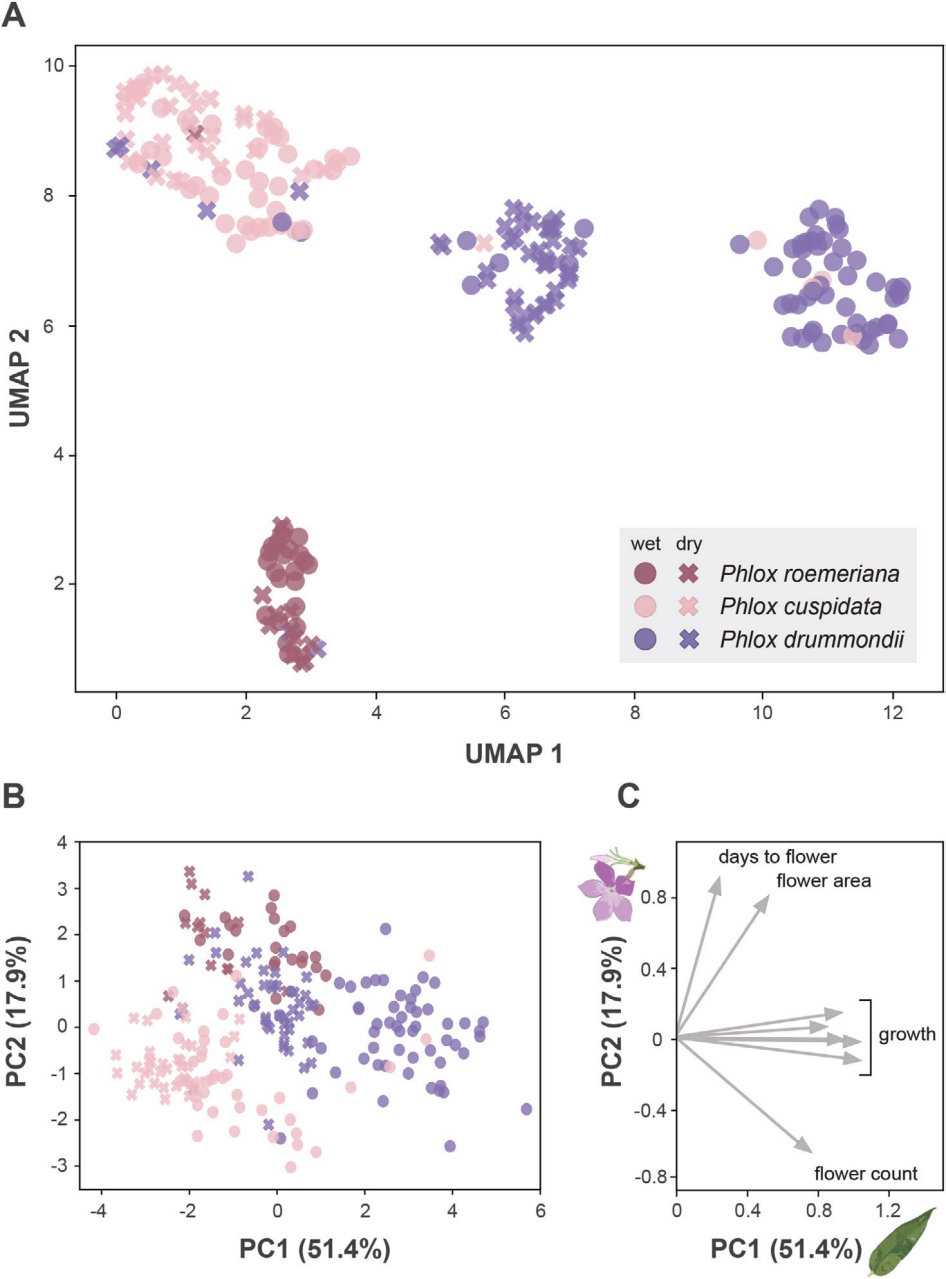
Multivariate responses to drydown experiment of annual *Phlox* species. (A) UMAP plot of *Phlox cuspidata* , *Phlox drummondii* , and *Phlox roemeriana* individuals based on measurements taken during the drydown experiment, colored by species, and marked by treatment. Dimensionality reduction was performed on mean corolla length, mean flower area, height, mean leaf length, leaf count, biomass (fresh and dry), days to flower, and flower count. (B) Plot of the first two principal components for *P. drummondii* , *P. cuspidata* , and *P. roemeriana* individuals based on measurements taken during the drydown experiment, colored by species, and marked by treatment. (C) PCA loading arrows for all drydown variables with a loading > |0.5| for PC1 and PC2. PC1 tends to reflect overall size and vegetative investment and PC2 tends to reflect floral investment (Table S10).

The three Texas annual *Phlox* species have distinct phenotypes, as evidenced by both the UMAP dimensionality reduction (Figure 3A) and the results from the principal component analysis (Figure 3B). The PCA loadings indicate that the primary contributors to PC1 are mean corolla length, height, mean leaf length, fresh and dry biomass (Figure 3C; Table S11). *P. cuspidata* is the smallest of the three species with narrow leaves, small flowers, and short stature, while *P. drummondii* is the tallest with wide leaves, and a robust growth form. PC2 is explained by variation in flower area and days to flower and is negatively associated with flower count (Figure 3C; Table S11). These traits tend to reflect floral investment associated with sexual reproduction. *P. roemeriana* has characteristically larger flowers and a distinct inflorescence structure with fewer flowers per inflorescence and therefore it is unsurprising that this species separates from the other two species along PC2.

Although all the species responded to water treatment, *P. drummondii* individuals responded with the greatest plasticity. This is observed in the UMAP dimensionality reduction on phenotypic traits which shows that *P. drummondii* has separate wet and dry clusters. In the PCA, the wet environment plants tend to have higher PC1 values compared to the dry environment plants for each species and this pattern is particularly robust for *P. drummondii* (Figure 3B).

#### 3.3.2 Univariate Response to Water Availability Across Species

**FIGURE 4:**
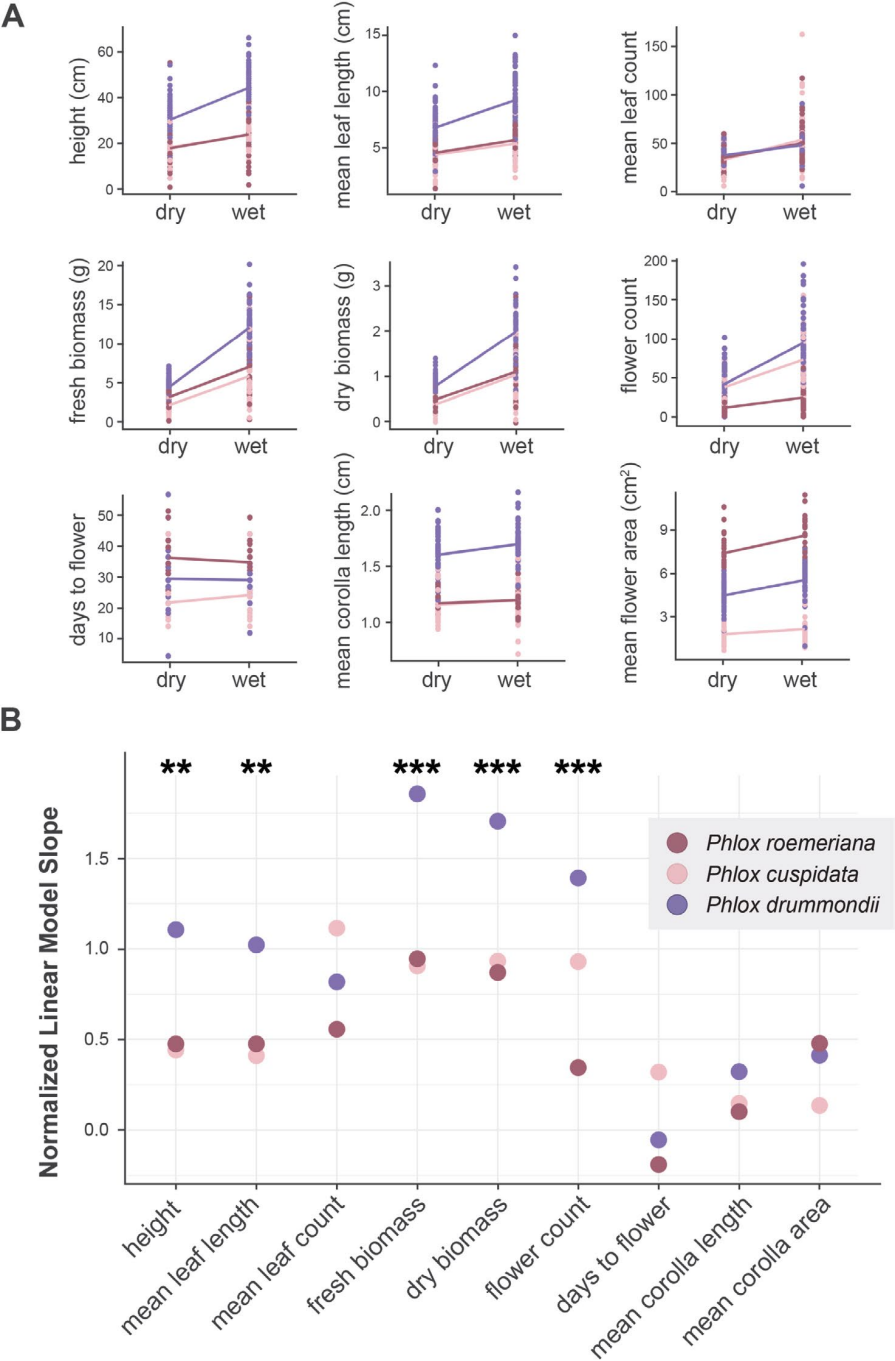
Univariate responses to drydown experiment of annual *Phlox* species. (A) Reaction norms for the drydown experiment, separated by treatment and species. Lines are from a simple linear regression and only present for visualization, not analysis. (B) Slopes of the linear mixed models for each species and measured variable. Data was normalized by *z*‐score prior to plotting, in order to ensure comparable slopes. Stars indicate the significance of the species by treatment interaction, calculated with an ANOVA test on the linear mixed models with Bonferroni correction (**p < 0.01, ***p < 0.001).

Univariate analyses of phenotypic traits reveal broad differences between species, between treatments, and significant differences in the magnitude of reaction to the treatments between the three species (treatment by species interaction) (Figure 4A). Nearly all traits varied either between species, treatment, or both with only leaf count showing no significant differences. Across treatments, the rank order of species for each trait did not change (Figure 4A,B). In general, *P. drummondii* is taller, has longer leaves, more biomass, and longer corollas than the other two species. The three species differ significantly in flower area with *P. roemeriana* having the largest flowers and *P. cuspidata* having the smallest flowers.

While the species are generally bigger with more flowers under wet conditions, the magnitude of this response was different across species as indicated by significant species by treatment interactions (*p* < 0.05; ANOVA on linear mixed models with Bonferroni correction) for height, mean leaf length, fresh biomass, dry biomass, and flower count (Figure 4A,B; Tables 2 and 3; Figure S2; Table S12). For all traits where the species responded differently to wet and dry conditions, *P. drummondii* has a steeper reaction norm slope than *P. cuspidata* or *P. roemeriana* (Figure 4B; Table 3; Table S12). In general, the three species tend to have more similar trait values under dry conditions with *P. drummondii* showing the greatest growth response to abundant water availability.

**TABLE 2:** Analysis of Variance (ANOVA) results from linear mixed models testing fixed effects of species, treatment, and their interaction on morphological and phenological traits.

Trait	Species			Treatment			Species–treatment		
	df	*χ* ^2^	Adj. *p*	df	*χ* ^2^	Adj. *p*	df	*χ* ^2^	Adj. *p*
Height	2	25.014	**< 0.001**	1	79.275	**< 0.001**	2	17.772	**0.001**
Leaf length	2	35.305	**< 0.001**	1	63.998	**< 0.001**	2	13.756	**0.009**
Leaf count	2	0.204	1	1	61.9	**< 0.001**	2	3.9544	1
Fresh biomass	2	92.108	**< 0.001**	1	339.046	**< 0.001**	2	50.569	**< 0.001**
Dry biomass	2	58.199	**< 0.001**	1	245.256	**< 0.001**	2	29.914	**< 0.001**
Flower count	2	93.739	**< 0.001**	1	115.087	**< 0.001**	2	26.349	**< 0.001**
Days to flower	2	59.2564	**< 0.001**	1	0.0787	1	2	4.4087	0.992
Corolla length	2	113.6758	**< 0.001**	1	5.3112	0.1907	2	1.5072	1
Flower area	2	171.4074	**< 0.001**	1	32.6098	**< 0.001**	2	6.9286	0.281

*Note:* For each response variable, the degrees of freedom (df), chi‐squared test value (*χ* ^2^) and associated *p*‐values are shown for the fixed effects of species, treatment, and species‐by‐treatment interaction. Bolded *p*‐values indicate significance.

**TABLE 3:** Results of Tukey's HSD test comparing pairwise species differences under dry and wet treatments.

Trait	Contrast	Dry		Wet		Contrast	Within species	
		Estimate	*p*	Estimate	*p*		Estimate	*p*
Height	C‐D	−8.536	0.3479	−16.174	0.17	**Dry C‐Wet C**	**−5.967**	**0.0098**
	C‐R	4.681	0.9594	5.120	0.9154	**Dry D‐Wet D**	**−13.605**	**< 0.0001**
	**D‐R**	**13.216**	**0.045**	**21.294**	**0.0001**	Dry R‐Wet R	−5.527	0.1224
Leaf length	C‐D	−2.147	0.0315	−3.588	0.1	Dry C‐Wet C	−0.975	0.119
	C‐R	−0.066	1	−0.228	1	**Dry D‐Wet D**	**−2.417**	**< 0.0001**
	D‐R	2.081	0.848	3.360	0.5	Dry R‐Wet R	−1.138	0.1258
Leaf count	C‐D	−5.226	0.9991	−0.596	1	**Dry C‐Wet C**	**−21.788**	**< 0.0001**
	C‐R	−1.190	1	9.120	0.9529	**Dry D‐Wet D**	**−17.158**	**< 0.0001**
	D‐R	4.036	1	9.716	0.9435	Dry R‐Wet R	−11.479	0.2449
Fresh biomass	**C‐D**	**−2.275**	**0.0409**	**−6.115**	**< 0.0001**	**Dry C‐Wet C**	**−3.607**	**< 0.0001**
	C‐R	−0.911	0.9737	−1.079	0.9019	**Dry D‐Wet D**	**−7.446**	**< 0.0001**
	D‐R	1.364	0.6913	**5.036**	**< 0.0001**	**Dry R‐Wet R**	**−3.775**	**< 0.0001**
Dry biomass	C‐D	−0.423	0.0532	**−0.963**	**< 0.0001**	**Dry C‐Wet C**	**−0.638**	**< 0.0001**
	C‐R	−0.094	0.9999	−0.055	1	**Dry D‐Wet D**	**−1.178**	**< 0.0001**
	D‐R	0.329	0.3813	**0.908**	**< 0.0001**	**Dry R‐Wet R**	**−0.599**	**< 0.0001**
Flower count	C‐D	−5.381	0.9989	**−22.959**	**0.0315**	**Dry C‐Wet C**	**−35.019**	**< 0.0001**
	**C‐R**	**25.541**	**0.0304**	**47.414**	**< 0.0001**	**Dry D‐Wet D**	**−52.598**	**< 0.0001**
	**D‐R**	**30.922**	**0.0031**	**70.373**	**< 0.0001**	Dry R‐Wet R	−13.146	0.62
Days to flower	**C‐D**	**−6.405**	**0.0093**	−3.605	0.443	Dry C‐Wet C	−2.392	0.6891
	**C‐R**	**−12.846**	**< 0.0001**	**−9.065**	**0.0002**	Dry D‐Wet D	0.409	1
	**D‐R**	**−6.440**	**0.0222**	−5.461	0.0836	Dry R‐Wet R	1.389	0.9985
Corolla length	**C‐D**	**−0.413**	**< 0.0001**	**−0.460**	**< 0.0001**	Dry C‐Wet C	−0.044	0.9843
	C‐R	−0.008	1	0.004	1	Dry D‐Wet D	−0.091	0.1232
	**D‐R**	**0.405**	**< 0.0001**	**0.464**	**< 0.0001**	Dry R‐Wet R	−0.032	1
Flower area	**C‐D**	**−1.824**	**0.0141**	**−2.643**	**0.0001**	Dry C‐Wet C	−0.309	0.9911
	**C‐R**	**−4.882**	**< 0.0001**	**−5.786**	**< 0.0001**	**Dry D‐Wet D**	**−1.128**	**0.0001**
	**D‐R**	**−3.058**	**0.0001**	**−3.143**	**< 0.0001**	**Dry R‐Wet R**	**−1.213**	**0.0141**

*Note:* Estimates and *p*‐values are shown for each trait comparison between species pairs: *Phlox cuspidata* vs. *Phlox drummondii* (C‐D), *Phlox cuspidata* vs. *Phlox roemeriana* (C‐R), and *Phlox drummondii* vs. *Phlox roemeriana* (D‐R). Bolded *p*‐values indicate significance (*p* < 0.05). For the full table including standard errors, degrees of freedom, and *t‐*statistics, see Table S12.

**FIGURE 5:**
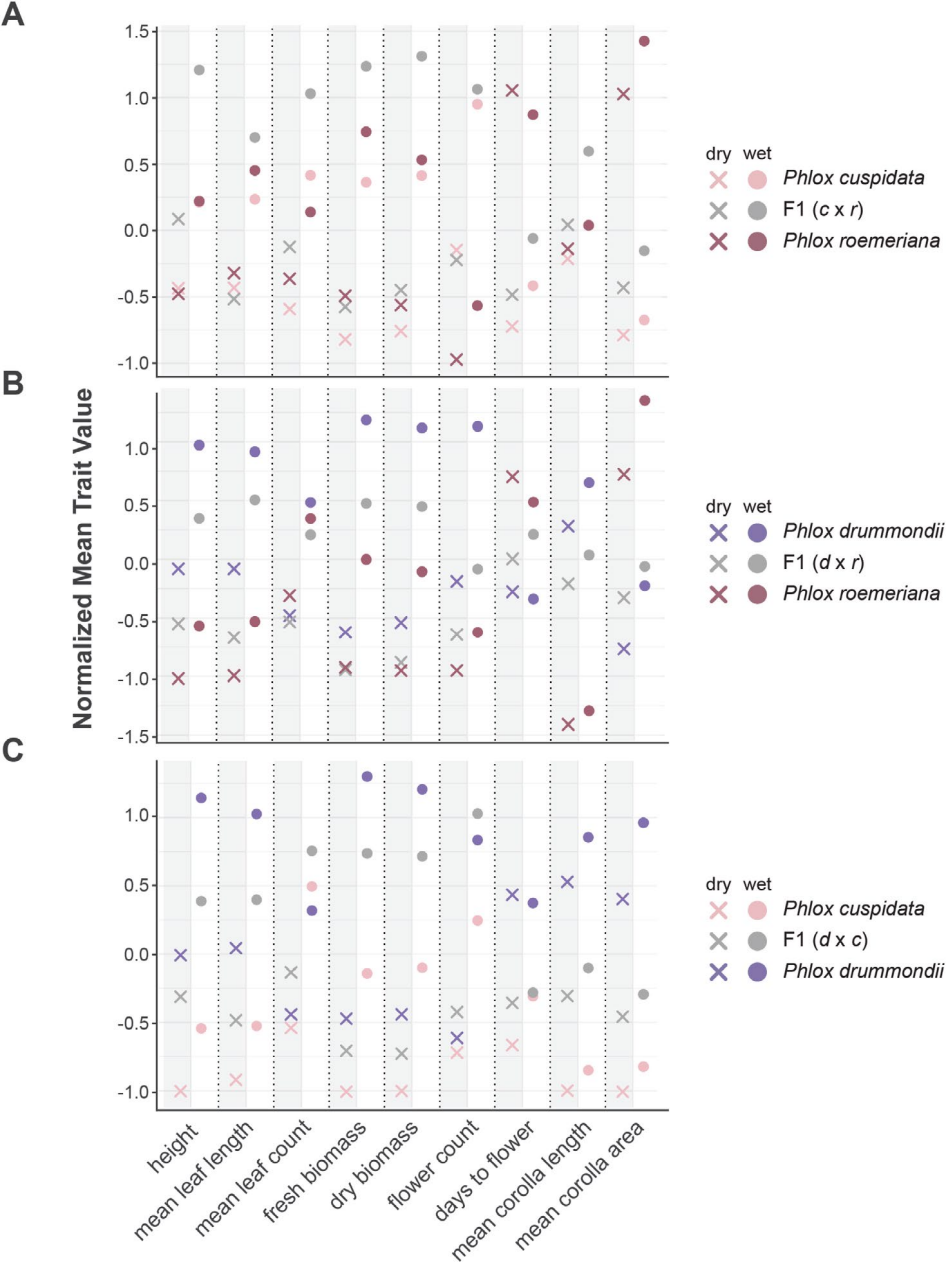
Differences in trait means of parental species and pairwise hybrids in response to drought. (A) Mean trait values for *P. drummondii* , *P. roemeriana* , and their F1 hybrids in wet and dry conditions. (B) Mean trait values for *P. cuspidata* , *P. roemeriana* , and their F1 hybrids. (C) Mean trait values for *P. cuspidata* , *P. drummondii* , and their F1 hybrids. All data *z*‐score adjusted.

Hybrids showed differential responses to water limitation. The two species that tended to be the most similar and had the weakest response to the treatment, *P. cuspidata* and *P. roemeriana* , had transgressive phenotypes in their hybrids. *P. cuspidata–P. roemeriana* hybrids were larger than their parents in most traits (Figure 5A; Tables 2 and 3; Table S12), particularly for the aforementioned vegetative growth traits. Trait values were particularly transgressive in the wet condition. In contrast, *P. drummondii–P. roemeriana* and *P. cuspidata–P. drummondii* hybrids were consistently intermediate between that of their parents (Figure 5B,C), a trend that persisted in both wet and dry conditions. When a principal component analysis was performed and the traits were not taken in isolation, the F1 hybrids of each of the species similarly fell in between their parents along the first two PCs (Figure S3), especially in the wet condition. In sum, the hybrids with *P. drummondii* parentage had consistent intermediate phenotypes in contrast to the transgressive phenotypes seen in *P. cuspidata–P. roemeriana* hybrids.

## 4 Discussion

We found that the clade of annual *Phlox* species native to the hot and dry environments of Texas occupies distinct ecological niches and expresses divergent phenotypes in response to water availability. Despite their close evolutionary relationships, these species differ in both their environmental associations and their patterns of phenotypic plasticity. Hybrids provided further insight into the genetic basis of these differences, where crosses involving the parent with the most plastic response ( *P. drummondii* ) produced consistently intermediate phenotypes, suggesting an additive genetic basis to many trait differences. On the other hand, *P. cuspidata–P. roemeriana* hybrids showed transgressive phenotypes. These contrasting hybrid responses underscore how divergence in plasticity and trait means can reflect different underlying genetic architectures. Together, our findings highlight how closely related lineages can evolve distinct ecological strategies and plastic responses, and how hybridization can reveal the genetic contributions to trait divergence in drought adaptation.

The different suitable ecological niches inferred for each of the species align with described ecoregions in Texas along an east–west gradient from the Gulf Coast Prairies and Marshes to the East Central Texas Prairies, to the Edwards Plateau (Griffith et al. 2004). Ecological differentiation among the three species is primarily driven by variation in precipitation patterns and soil moisture. The major axis of environmental variation distinguishes species based on water availability, with *P. roemeriana* occupying the coolest, driest habitats, *P. cuspidata* restricted to warmer, wetter sites, and *P. drummondii* occurring in intermediate habitats. Notably, soil properties are likely to interact with precipitation to influence soil moisture availability (Sehler et al. 2020; Suliman et al. 2024; Rajak et al. 2025), potentially explaining why *P. drummondii* habitats exhibit the lowest soil moisture despite intermediate precipitation levels. The niche breadth of *P. cuspidata* was notably the greatest in this clade, likely because it is the only selfing species. Selfing is thought to facilitate colonization of novel and oftentimes harsh habitats by removing mate limitation and being associated with rapid life cycling (Snell and Aarssen 2005; Busch and Schoen 2008; Razanajatovo et al. 2016; Gorman et al. 2020). Across 14 species of *Collinsia* and *Tonella* (Plantaginaceae), selfing species exhibit significantly wider niche breadths than their outcrossing sister species (Grant and Kalisz 2020). However, such advantages may be transient, as genomic homozygosity is expected to reduce long‐term persistence (Park et al. 2018). Consistent with its specialization to xeric limestone habitats (Erbe and Turner 1962; Levin 1978a), *P. roemeriana* exhibited the narrowest niche breadth.

Trait differences in these *Phlox* do not align neatly with expectations based on soil water availability. Plants adapted to low water availability tend to have small leaf area, less biomass, and earlier flowering as part of a drought escape strategy (Chaves et al. 2003; Exposito‐Alonso et al. 2018). Under normal greenhouse conditions, *P. cuspidata* displayed the lowest biomass, narrowest leaves, smallest flowers, and earliest flowering time, despite occurring in the wettest soils with the highest precipitation. These traits align with its selfing mating system and the suite of characteristics commonly associated with the selfing syndrome (Ornduff 1969; Sicard and Lenhard 2011; Cutter 2019). In contrast, *P. drummondii* had the most substantial biomass, biggest leaves, and tallest stature, despite occupying dry habitats with the driest soils. *P. roemeriana* , which occurs in the hottest sites with the lowest levels of precipitation, produced large flowers with a distinctive inflorescence but had intermediate vegetative size.

Taken together, these patterns suggest that vegetative traits may be shaped by selective pressures beyond water availability alone. In particular, plants restricted to xeric limestone or other nutrient‐poor soils may experience strong selection on growth form and resource allocation, such as in native lineages of *Phragmites australis* , which are low‐nutrient specialists with smaller leaves and biomass but more efficient photosynthetic mechanisms relative to invasive lineages (Mozdzer and Zieman 2010). Investigating physiological traits such as specific leaf area, photosynthetic rate, and biomass accumulation both in the field and under controlled conditions will therefore be important for testing how adaptation to nutrient‐poor limestone habitats and the selfing syndrome influence vegetative trait evolution in these species. Incorporating genetic data alongside reciprocal transplants both within and between species could also provide critical insight into whether these phenotypes confer adaptive advantages in their native habitats and whether drought stress does occur. For example, research on *Epidendrum fulgens* (Orchidaceae) incorporating microsatellite markers, ecological niche modeling, and phenotypic data found that despite niche differentiation, genetic structure did not constrain ecotypic differentiation, indicating that local adaptation can occur independently of population genetic structure (de Lima et al. 2024). Ultimately, combining physiological, ecological, and genetic approaches will help clarify how nutrient limitation, mating system, and genetic architecture interact to drive trait divergence and adaptive potential in *Phlox*.

All three species exhibited substantial phenotypic plasticity in response to water availability, consistently reducing vegetative size, biomass, and leaf production under drought. In line with Berg's hypothesis of floral homeostasis (Berg 1960), floral traits showed limited plasticity, and neither flowering time nor corolla tube length changed significantly across treatments. This decoupling of vegetative and floral trait plasticity has been reported across a range of systems and environmental contexts, for example in *Arabidopsis thaliana* under altered light conditions (Brock and Weinig 2007), in *Campanula rotundifolia* across temperature treatments (Pélabon et al. 2013), and in *Plantago lanceolata* under varying water and light regimes (Villellas et al. 2021). Evolutionary theory predicts that traits most critical for reproduction evolve stronger genetic differentiation and exhibit less environmental plasticity (Stearns and Kawecki 1994). Our results are consistent with this pattern, as floral traits tend to be a key determinant of fitness in short‐lived plants.

In line with general plant responses to drought, these annual *Phlox* species reduce biomass and leaf size under limited water availability, with notable differences among species. Across the three species we find plasticity corresponds to a major shift in morphological space from wet to dry along the growth axis PC1. This axis also corresponds to significant differentiation between the three species but does not correspond to the associated niche differentiation inferred between the species. Specifically, *P. cuspidata* occupies the wettest habitat and yet has the most negative values along PC1 in morphological space which is associated with a plastic response to dry conditions, while *P. drummondii* occupies the driest soils in its native range but morphologically occupies the most positive PC1 values corresponding to well‐watered phenotypes. This pattern suggests that the direction of phenotypic plasticity does not match the direction of phenotypic divergence. Instead, this pattern could further indicate that broad patterns of vegetative and morphological divergence between the species are not predominantly driven by adaptation to water availability. In the case of *Phlox*, this plastic response did not correspond to a path of least resistance in ecological divergence (Fox et al. 2019; Liu et al. 2022). Similarly, patterns of morphological divergence do not appear to be the result of canalization of a plastic response after persistent selection (Agrawal 1999; Chun et al. 2007; Wund et al. 2008).

Among the three species, *P. drummondii* displayed the strongest plastic response to water availability. Under dry conditions, species converged on similar reduced vegetative display, while *P. drummondii* displayed a disproportionate release from drought and a subsequent shift in phenotype when under wet conditions relative to either *P. cuspidata* or *P. roemeriana* . Previous work hypothesizes that plasticity may be positively correlated with environmental niche breadth (Pfennig et al. 2010; Leung et al. 2020; Stotz et al. 2021). In the case of these *Phlox*, we find that *P. drummondii* occupies an intermediate habitat but does not inhabit the broadest environmental niche. Nevertheless, its ability to capitalize on high moisture environments suggests a flexible growth strategy constrained primarily by external water availability.

Finally, we found no evidence of intrinsic dysfunction among the three species, as all F1 hybrids exhibited vegetative and floral trait values intermediate to or exceeding those of their parental species. Notably, hybrids between *P. cuspidata* and *P. roemeriana* displayed transgressive traits, becoming larger than either parent, while hybrids with *P. drummondii* as a parent were typically intermediate. This finding is in contrast to Haldane's rule, which predicts that hybrid dysfunction should emerge as an indirect byproduct of divergent adaptation to differing environments (Haldane 1922). These results suggest that ecological divergence among these species has not generated intrinsic barriers severe enough to limit hybrid performance, at least in terms of growth, viability, and floral morphology. On the other hand, the rarity of hybrids in nature, together with strong evidence for reinforcing selection against hybridization in this system (Hopkins and Rausher 2012), suggests that limited fertility may constrain hybrid formation. To clarify this, future work should map hybrid sterility (i.e., pollen and ovule breakdown) to determine whether hybrids are developmentally functional (Araripe et al. 2010; Ouyang et al. 2010; Fishman et al. 2013).

Because our experiments also imposed water stress, these findings provide insight into how extrinsic factors may shape reproductive isolation. If hybrids are more vulnerable to water stress than parental taxa, then water limitation could represent a powerful ecological barrier that restricts hybrid establishment and maintains species boundaries (Holá et al. 2017; Otwani et al. 2023). However, we observed little evidence of reduced performance under drought. In fact, *P. cuspidata* × *P. roemeriana* hybrids even exceeded parental species in size, suggesting that hybridization could be advantageous under stressful conditions. It remains unclear; however, whether such novel phenotypes would be favored under natural stressful environments. Indeed, hybrids with novel character traits have been described as ecologically inviable across other systems (Brys et al. 2013; Melo et al. 2014; DiVittorio et al. 2020). Therefore, growing hybrids in parental locations would inform whether hybrid dysfunction arises primarily from extrinsic ecological factors, intrinsic developmental barriers, or their interaction. Nonetheless, our results suggest that hybridization in this clade may reinforce isolation where stress disproportionately disadvantages hybrid phenotypes, or alternatively generating ecological or phenotypic novelty that enables colonization of new or changing habitats.

Our findings reveal species‐specific patterns of growth and plasticity, suggesting potential links between ecology, evolutionary history, and phenotypic plasticity in shaping patterns of divergence in annual *Phlox*. These results underscore the importance of environmental context in maintaining species differences and illustrate how plastic responses can contribute to the ecological dynamics of closely related species.

## Conflicts of Interest

The authors declare no conflicts of interest.

## Supporting information


**Appendix S1:** pei370098‐sup‐0001‐AppendixS1.docx.

## Data Availability

The data that support the findings of this study are openly available in Dryad at doi: https://doi.org/10.5061/dryad.x3ffbg7z4.
